# Structural and physical properties of the dust particles in Qatar and their influence on the PV panel performance

**DOI:** 10.1038/srep31467

**Published:** 2016-08-16

**Authors:** Brahim Aïssa, Rima J. Isaifan, Vinod E. Madhavan, Amir A. Abdallah

**Affiliations:** 1 Qatar Environment and Energy Research Institute, Hamad Bin Khalifa University, Qatar Foundation, P.O. Box 5825, Doha, Qatar; 2 College of Science and Engineering, Hamad Bin Khalifa University, Qatar Foundation, P.O. Box 5825, Doha, Qatar

## Abstract

Recently, extensive R&D has been conducted, both by industry and academia, to significantly raise the conversion efficiency of commercial photovoltaic (PV) modules. The installation of PV systems aimed at optimizing solar energy yield is primarily dictated by its geographic location and installation design to maximize solar exposure. However, even when these characteristics have been addressed appropriately, there are other factors that adversely affect the performance of PV systems, namely the temperature-induced voltage decrease leading to a PV power loss, and the dust accumulation (soiling). The latter is the lesser acknowledged factor that significantly influences the performance of PV installations especially in the Middle East region. In this paper we report on the investigation of the structural and physical properties of the desert-dust particles in the State of Qatar. The dust particles were collected directly from the PV panels installed in desert environment and characterized by different techniques, including scanning electron, optical and atomic force microscopies, X-ray diffraction, energy-dispersive, UV-Vis, micro-Raman and Fourier transform infrared spectroscopy. The vibrating sample magnetometry analyses were also conducted to study the magnetic properties of the dust particles. The influence of the dust accumulation on the PV panel performance was also presented and discussed.

The economic development has always been intimately correlated with increasing energy use and growth. Renewable energy can help decouple that correlation, contributing thereby to sustainable development. In this context, photovoltaic (PV) solar energy conversion is a clean and practical technology with huge potential. Solar PV technology is well-proven for producing electricity, where the global production has been increasing 370 times than that in 1992^1^. However, it is not yet widely deployed; PV efficiency optimization and cost reductions could significantly expedite the uptake of novel solar technologies. Moreover, for an optimal management and analysis of expected PV performance, the location sites become increasingly important.

Some of the main challenges that face the deployment of solar energy in large scale scheme in the Middle East (ME) region are the high operating temperature and dust accumulation on PV modules. While both effects result in a reduction of the kWh generated by the solar panels, focus was put in the current study on the investigation of the soiling effect, since geographically, the ME region is frequently affected by sand storms and characterized by a high dust concentration^2^.

The PV power losses due to the dust accumulation can be significant, particularly, in areas similar to Qatar climatic conditions, characterized by an arid nature which includes high air humidity and patchy rainfalls, making dust accumulation highly problematic^3^,^4^. Dust deposition on solar modules depends on several factors; the tilt angle of the solar module, exposure period, site climate conditions (temperature, humidity, wind speed and direction and dust properties).

Dust accumulation is mainly affecting the optical properties of the PV modules, which results in decreasing their generated photocurrent output. It is then of primary importance to quantify the effect of soiling by measuring the corresponding optical losses. Several relevant studies have been already published in the investigation of the effect of soiling on the optical properties of photovoltaic glass in different areas in the world^5^,^6^,^7^. In the ME region, Mani and Pillai^2^ have reported in their review article that as high as 17% of the PV power was lost due to dust deposition on PV modules in Kuwait city (state of Kuwait), after only six days of dust exposure. Sayigh *et al*.^8^ have conducted a similar study in the same region as a function of the tilted angles, and reported a reduction in the optical transmittance from 64 to 17% when the tilt angle changes from 0^o^ to 60^o^, respectively. Besides, in the city of Riyadh (Saudi Arabia (KSA)), various studies have been conducted, where Salim *et al*.^9^ have indicated that eight months of solar cell exposure to desert conditions have caused 32% reduction in the PV module efficiency, while Sayigh *et al*.^10^ have revealed a power loss of about 11.5% for only 72 hours exposure.

However, it is worth mentioning at this level that dust characteristics are location-specific since they depend mainly on various meteorological parameters, such as temperature, humidity, wind speed and direction, and installation factors that include tilt angle and surface properties. Moreover, additional variations in the morphological, structural and more generally the physical characteristics of the dust particles could be also location dependent. This in turn results in clear disparities in terms of the soiling effect on the PV power output with respect to the geographical location of the PV panels.

Since the processes of dust are complex, it requires thorough investigation of the characteristics of the dust, taken from the same locations of PV solar panels. In this paper, we report on the structural, optical and magnetic properties of the dust particles collected directly from solar modules installed at the Solar Test Facility in Doha (State of Qatar). Dust samples are characterized by scanning electron microscopy (SEM) and optical microscopy to determine the structural properties and particle size distribution. X-ray diffraction (XRD) and energy-dispersive spectroscopy (EDS) are used to study the crystal orientation and elemental composition, respectively. Optical properties are measured using UV-Vis spectroscopy; while microRaman and FTIR are performed to detect the presence of specific compounds such as minerals. The vibrating sample magnetometry analysis is conducted to evaluate the magnetic properties of the dust particles. Finally, the influence of the dust accumulation on the photovoltaic performance is also discussed by highlighting its effect on the current-voltage (IV) curve characteristics. This study represents a pioneer work on a thorough characterization of dust particles in the state of Qatar which is an essential step towards an in-depth investigation of a suitable dust mitigation protocol and efficient PV deployment in this region.

## Experimental

**Figure 1:**
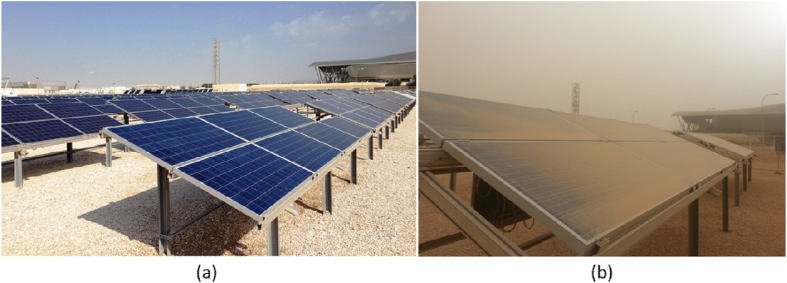
The Solar Test Facility located at the Science and Technology Park, in Doha (State of Qatar) with different PV technologies installed to study the performance and reliability of PV in Qatar climatic conditions; (**a**) PV modules after scheduled cleaning and (**b**) PV modules after dust storm inducing soiling.

Different PV technologies (including crystalline silicon and thin films) were installed at the Solar Test Facility located in Doha (State of Qatar) with a total of 150 kW power production capacity (Fig. 1). The data acquisition system provides IV characteristics of PV modules under real operating conditions. From each array, the direct (DC) and alternative (AC) current output power and module temperature were monitored on one minute time interval. The metrological station provides Direct Normal Irradiance (DNI), Diffused Horizontal Irradiance (DHI) and Global Horizontal Irradiance (GHI) in intervals of one minute real-time data of the three solar irradiation components. The global solar irradiation of the module on a fixed 22° tilt angle due to south (Plan of Array G-POA), ambient air temperature, cell temperature, wind speed and direction were also monitored continuously.

Borosilicate plate glass samples with dimensions of 25 mm × 10 mm × 2 mm (width × length × thickness) were used as work pieces. To investigate the effects of dust on the surface characteristics of the glass substrates, actual dust accumulation was performed in real environment. The dust particles were collected directly from PV panels installed at the Solar Test Facility. Morphological characterization of the dust was performed using SEM equipped with EDS (Jeol-JSM-6300F at 10 kV accelerating voltage without any conductive coating) and AFM (NanoScope III, Digital Instrument, operated in contact mode at room temperature in ambient air). The particle size distribution was obtained by measuring 200 dust particles. Structural studies were performed using XRD (Bruker D8–40 kV/40 Ma generator-9 Advanced diffractometer, with a Cu-K_α_ radiation source, λ = 1.5406 Å). The typical settings of the XRD instrument were as follows: 40 kV and 30 mA for the x-ray source and a scanning angle (2θ) range of 5°–90°. The optical transmittance was measured using a UV-Vis spectrometer (Jenway-67 Series spectrophotometer) and FTIR (FTIR-Thermo Nicolet Model 6700) spectra were collected in the wave number range of 400 to 4000 cm^−1^. The microRaman measurements were performed at room temperature, with the 532 nm (2.49 eV) laser radiation of an Ar^+^ laser focused onto the sample with a spot size of 1 μm and a power normally not exceeding 5 mW (LabRAM HR system, equipped with a CCD detector). Measurement data were collected in a backscattering geometry in the range of 100–2000 cm^−1^ with a spectral resolution of 0.5 cm^−1^.

The optical analysis of the dust particles deposited on glass substrates was performed using Olympus (IX73) optical microscope. The objective lens of 40X magnification was used. The optical images were processed and analyzed by CellSens software (Olympus Corporation, USA).

To measure the magnetic properties of the sample dust, we used the Vibrating Sample Magnetometer (VSM) to obtain the full hysteresis loop (MicroSense^TM^, Model 10 VSM). To accurately measure the magnetic properties, only small sample can be measured which must be fixed on the support rod coming from the vibrating unit. The dust of the sample must be contained to avoid dust dispersion in the equipment. The dust was initially placed on a small tape support (5 mm by 5 mm) then two pieces were folded over the dust (in perpendicular directions). The result gives a piece a tape of 1 cm by 1 cm of about 80 mg in which a small amount of about 10 mg of dust is confined.

## Results and Discussion

**Figure 2:**
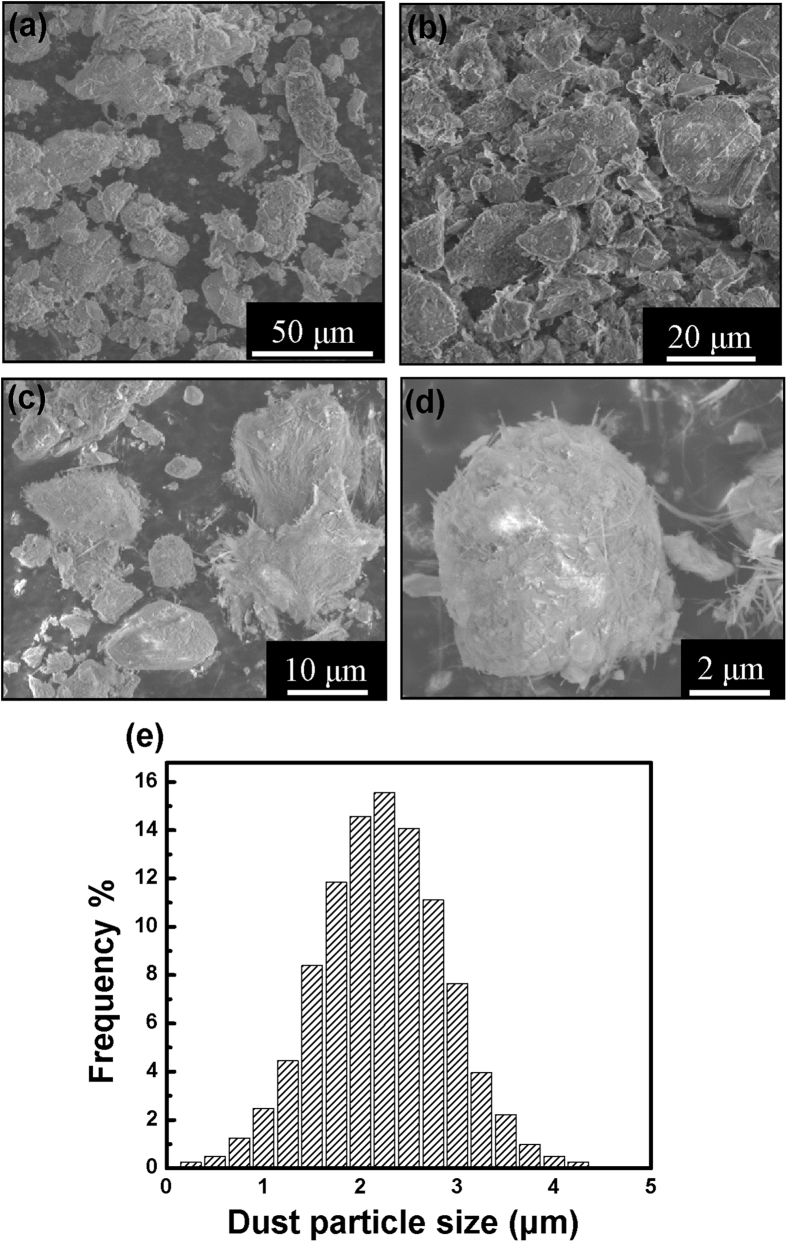
(**a–d**) Representative SEM micrographs with various magnifications of the desert-dust particles collected directly from the PV panels located at the Solar Test Facility (Doha, Qatar). (**e**) Particle size distribution histogram is shown in (**e**).

**Figure 3:**
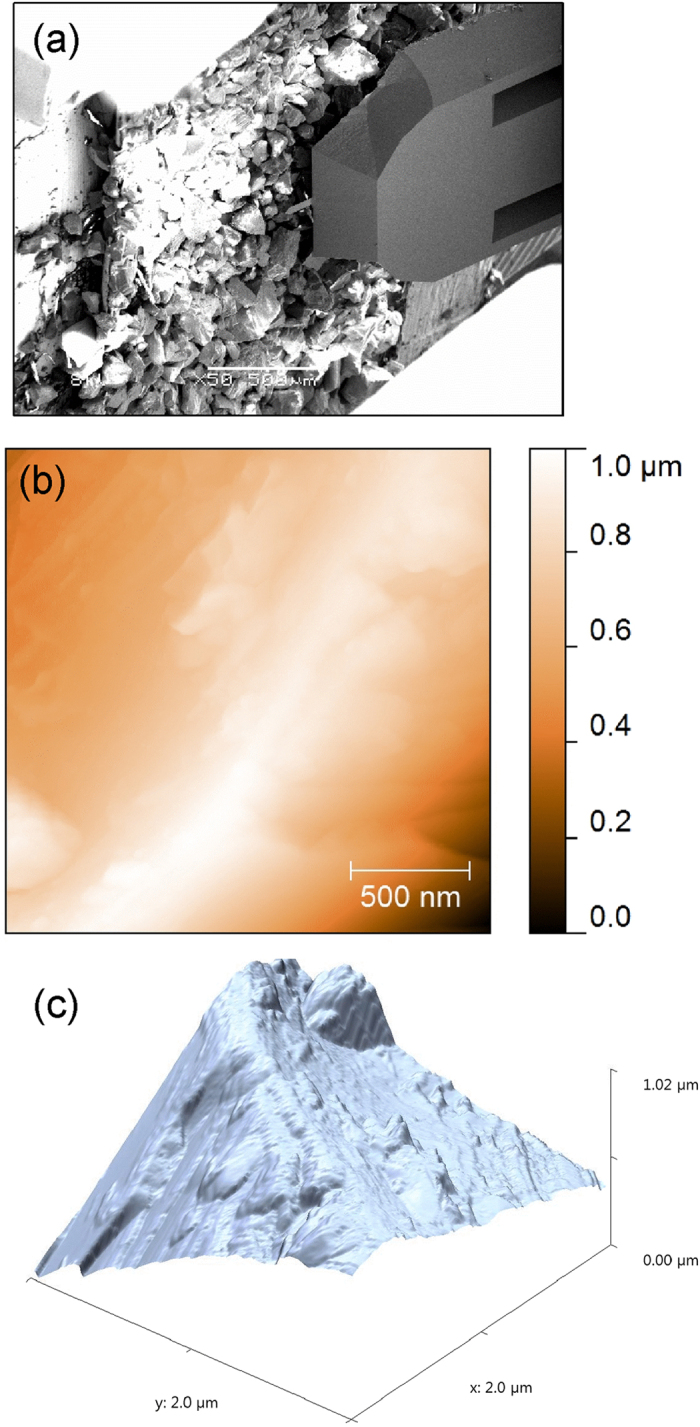
(**a**) SEM image of the AFM cantilever approaching the dust particles, (**b**) Atomic force micrograph of dust particle taken in the contact mode, and (**c**) its corresponding three-dimensional representation.

Figure 2a shows the representative SEM micrographs of dust particles taken at different magnification, where various particle sizes, with different morphologies are seen. The Gaussian distribution of the particles sizes was found to be centered at around 2 μm (Fig. 2b), however, larger particles in the order of few tens μm diameter are also observed. Although, some particles have irregular shapes, the majority of dust particles are rather spherical-like. Figure 3 shows the AFM contact mode image taken on a single dust particle, with a 3 dimensional average size of 2 μm.

With further analysis of the images of the dust particles, two principle quantities were identified; the aspect ratio (equation 1) and the shape factor (equation 2) defined as:

The aspect ratio


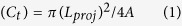


where *A* is the cross-sectional area, and *L*_*proj*_ is the longest projection length of the dust particle^5^.

The shape factor


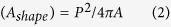


where P is the perimeter of the dust particle^5^.

The particle diameter and the area can be generated from the SEM characterizations. The diameter of a circle with equivalent area is considered for spherical dust particles; whereas, an ellipse particle shape model can be used by assuming the longest projection as the major axis and preserving the cross-sectional area of the particle for non-spherical shaped dust^5^.

The aspect ratio is related to the particle roundness and approximately represents the ratio of the major axis to the minor axis of the ellipsoid best fit to the particle. In addition, the shape factor is the inverse of the particle circularity, which is associated with the complexity of the particle. In this case, a shape factor of unity corresponds obviously to a perfect circle. The aspect ratio and the shape factor are found to change with the particle size in a no linear relation, however, a general correlation states that the particle aspect radio decreases with increasing particle size, while the shape factor increases with increasing particle size. In our study, the shape factor approaches unity for the smaller particles as they are spherical in shape, while for the large longitudinal particles, the median shape factor almost reaches 3.5. In comparison with other studies, a value of 3 was reported both for the dust samples collected in Saudi Arabia^11^, and for samples from African Sahara collected over the tropical North Atlantic area^12^. The increase of aspect ratio indicates higher probability that the particles are aggregates, causing thereby a substantial increase in perimeter relative to their area.

**Figure 4:**
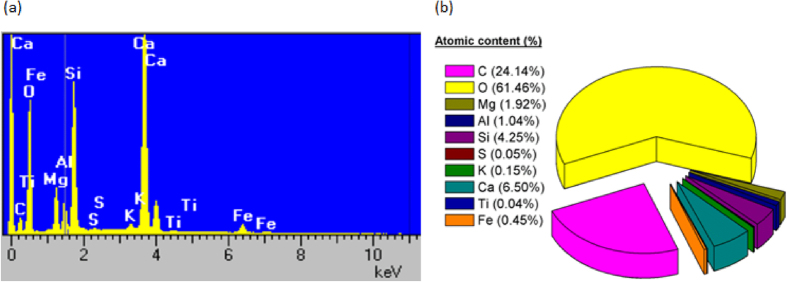
(**a**) Energy dispersive spectra of the dust particles, (**b**) the corresponding chemical composition of the dust particles.

Figure 4 presents the EDS data for the dust particles with their constituting chemical elements presented from highest to lowest atomic concentration. The results show that dust particles have different concentrations of non-uniformly distributed elements and compounds. It’s to underline that the quantitative atomic content of oxygen and carbon have to be taken with high care since EDS is not a suitable technique to probe these elements.

The presence of traces of potassium can be associated with sea salt as Qatar is located in the Arabian Gulf. Nevertheless, no chloride was detected either by EDS in our samples in contrast to what was reported by Yilbas *et al*.^5^ on the composition of dust particles collected in Saudi Arabia. However, the absence of both sodium and chloride are similar to the results reported by Reid *et al*.^13^ in their study of the African dust. Reid and coworkers mentioned that it is expected that sodium and chloride as sea salt, are independent species from dust^12^. When an aerosol sample is collected for analysis, the trace values of any sodium and chloride concentration in the dust will not be detectable compared with the presence of salt as dissolved in the compound form. This fact was supported as well by Yilbas *et al*.^5^ where they have reported that their EDS data did not support the molar ratio for NaCl, confirming thereby that the dust particles did not contain salt crystals.

The sulfur concentration is correlated with calcium in most studies^5^,^13^ which is associated with the formation of anhydrite or gypsum component (CaSO_4_). The ratio of Ca to Si in our study is found to be 1.53, whereas it was around 0.18 for African dust^12^ and 0.76 for dust particles collected in KSA^5^. In the latter case, the authors have interestingly revealed that different particle shapes are rich in different elements. As a matter of fact, quadrangular particles which were initially deformed from a cubic shape were rich in sodium and chlorine, while the aggregated particles were rich in calcium and oxygen. In comparison, flake-like particles were rich in calcium and silicon^5^. Changes in composition with changes in particle size and morphology have been reported previously^14^,^15^. Qi *et al*.^15^ have performed EDS analysis for bulk and single dust particles in three different locations in China, where silicon was found to be the most dominant element in all locations with an average concentration ranging between 33 to 46% based on their atomic content. In addition, they reported that coarse particles were rich in Al, Si and Fe, in comparison with particulate materials collected in the same locations which were richer in Mg, Cl and K.

**Figure 5:**
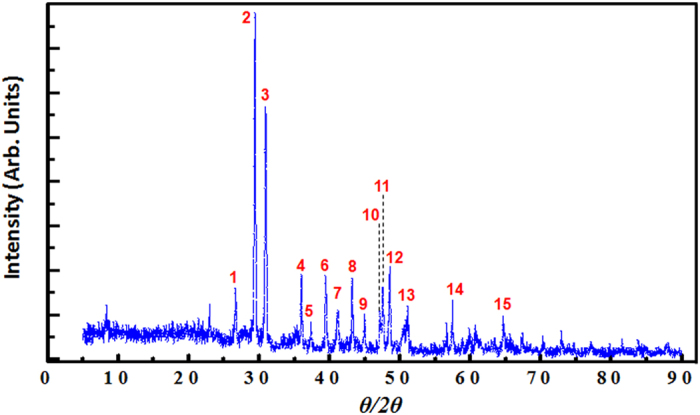
Typical X-ray diffraction pattern of dust particles in Qatar.

Figure 5 shows the X-ray diffractogram of the dust particles together with a summary of the main corresponding peaks as presented in Table 1. Qualitative analysis of the XRD data shows that dust particle are mainly composed of calcite (CaCO_3_), quartz (SiO_2_), sillimanite (Al_2_(SiO_4_)O), wuestite (FeO), olivine (Mg_2_(SiO_4_)) and akermanite (Ca_2_Mg(SiO_7_)).

Table 2 shows a summary of the quantitative analysis which reveals that 58% of the dust particles are composed of calcite.

Although Yilbas *et al*.^5^ have reported high content of calcite similar to our case (i.e., dust particles collected in Saudi Arabia versus those collected in Qatar), their dust particles were found to contain sodium chloride (as supported by their EDS analysis) which was not detected in our study. The presence of sodium and potassium peaks related to salts was explained by the proximity to the Arabian Gulf. Moreover, as the concentration of chlorine was found to vary for different dust particles, and as the EDS data do not satisfy the molar ratio for NaCl; authors concluded that the dust particles did not contain salt crystals as such; instead, NaCl was supposed to rather dissolve in the compound form. Peng *et al*.^16^ have supported similar assumptions, where they suggested that for dust particles of <10 μm, the potassium chloride and sodium chloride particles were partly covered by other materials which are insoluble in water and consisted mainly of iron oxides or salts containing calcium.

Moreover, sulfur was not a main component of dust particles collected in Qatar (0.05 atm. % only), while it was found with significant proportion in the dust of Saudi Arabia (around 2.4 atm. %). However, as mentioned earlier, the sulfur can be correlated with the calcium in the dust, such as the anhydrite or gypsum component (CaSO_4_). Besides, iron particles in Qatar dust are most probably due to the FeO while in those from Saudi Arabia they are highly likely related to clay-aggregated hematite (Fe_2_O_3_). In general, the difference in composition of dust particles is related to the source of those particles. However, since the solar test facility is located in an urban zone, surrounded by heavy traffic, it’s not excluded that, in addition the natural desert particles, part of the deposited dust (e.g. Ti traces observed in our case) could probably provide from contamination sources as from the re-suspension of dust produced by vehicular brake wear and asphalt, influencing thereby its chemical composition^17^,^18^.

**Figure 6:**
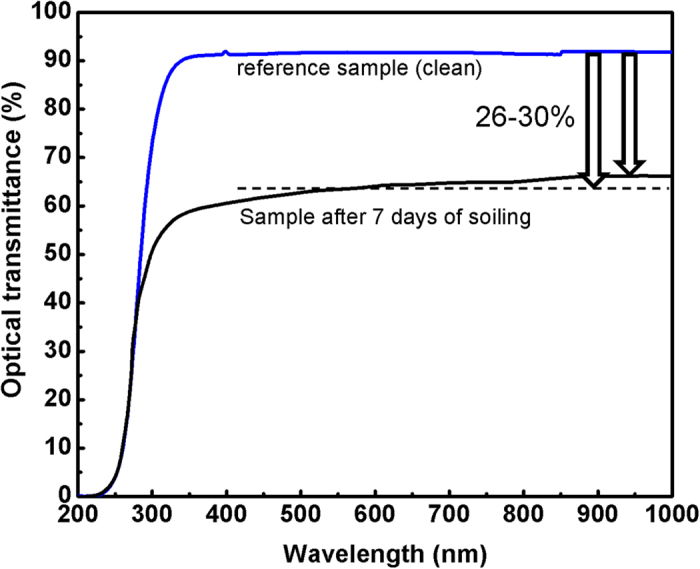
UV-Vis transmission spectrum of clean/dusty samples collected from the Test Facility site after 7 days of soiling.

UV-Vis transmission spectra of a dusty glass substrate compared with cleaned one is shown in Fig. 6. The dusty sample was collected from the Test Facility site after 7 days of soiling at zero angle inclination. The spectrum shows up to 30% reduction in the optical transmittance, decreasing from 90 to about 60%.

**Figure 7:**
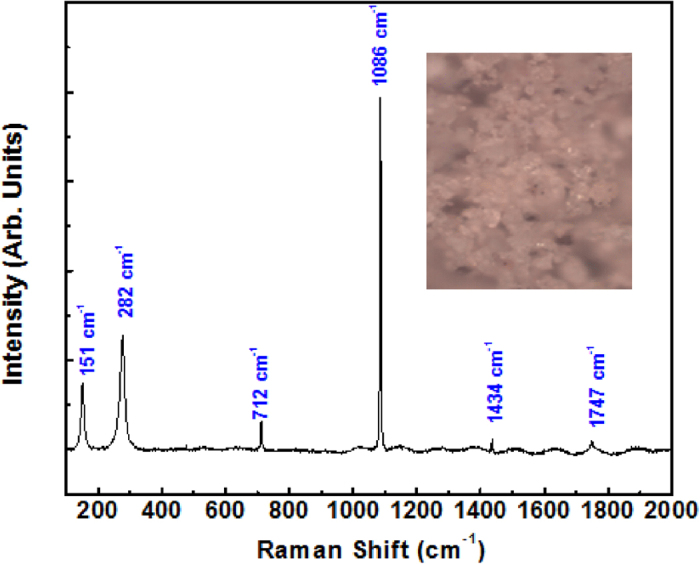
Raman spectra of the dust samples collected from the PV panels in the test site. The inset is an optical image of the probed zone.

The corresponding Raman spectrum of the dusty sample is recorded in the range of 100 to 3000 cm^−1^ and is displayed in Fig. 7. The spectrum shows characteristic peaks located at 151, 282, 712, 1086, 1434 and 1747 cm^−1^. These Raman peaks match very well with those related to the calcite CaCO_3_ mineral referring to symmetric A_1g_ vibration modes in calcite at 1086 cm^−1 ^19,20. In this mineral, the lattice mode vibrations are also reported at 151, 282, 712 cm^−1^ (in-plane bending) 1086 cm^−1^ (symmetric stretching), 1434 cm^−1^ (asymmetric stretching), and 1747 cm^−1^ (ʋ_1_ + ʋ_4_ overtone)^20^,^21^. The Raman spectra analysis confirms indeed that our dust structures contains calcite compounds and supports well the compositional information presented in the Table 2. However, these observations have to be taken with a certain care since this technique is rather qualitative (especially if one takes into account the small spot size of the probed zone ∼1 μm-diam.), moreover, the presence of Raman peaks related to akermanite, sillimanite and olivine minerals is not excluded, (as a matter of fact, the peak located at 712 cm^−1^ could be also characteristics of sillimanite composition^22^), and their associated Raman peaks could be simply too weak and/or embedded within the recorded Raman spectrum of the dominant species (i.e. calcite, 58% atm.%).

**Figure 8:**
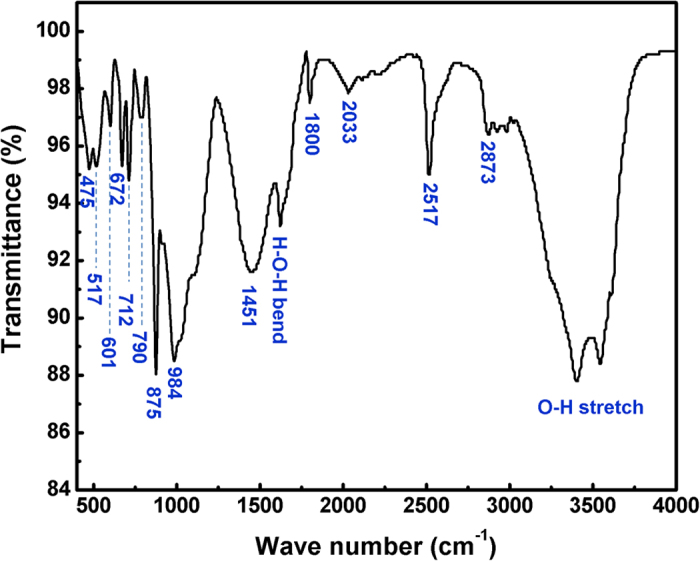
Typical FTIR spectra of the dust sample.

FTIR spectroscopy gives complementary information since it refers to the interaction between the molecular bonds structures. FTIR spectrum of the same dusty sample is recorded in the Mid-IR range (400–4000 cm^−1^) and is presented in Fig. 8.

The spectra shows various fundamental infrared vibration absorption bands corresponding to calcite mineral, located at 475, 517, 601, 672, 712, 790, 875, 984, 1451, 1800, 2033, 2517 and 2873 cm^−1^. The positions of these peaks are unique to a mineral structure and give identification of the mineralogy as follows: peaks at 712 cm^−1^ corresponds to ʋ_4_-symmetric CO_3_ deformation, the 875 cm^−1^ to ʋ_2_-Asymmetric CO_3_ deformation, the 1451 cm^−1^ to ʋ_3_-Asymmetric CO_3_ stretching, the 1800 cm^−1^ corresponds to ʋ_1_ + ʋ_4_ overtones and the 2517 cm^−1^ to 2ʋ_2_ + ʋ_4 _21. The peak found at 1086 cm^−1^ in Raman spectroscopy (Fig. 7) represents the ʋ_1_-Symmetric CO_3_ stretching, to ascertain the presence of calcite mineral structure^21^. The following peaks are both IR and Raman active: Raman modes detected at 712, 1434 and 1747 cm^−1^ in Fig. 7 are in agreement with the peaks at 712, 1451 and 1800 cm^−1^, respectively in the FTIR spectrum which correspond to an in-plane bending and/or to ʋ_4_-Symmetric CO_3_ deformation^21^. Peaks at 475, 517, 790 and 984 cm^−1^ are found in all limestone minerals^21^. Bands at 2033 and 2873 cm^−1^ could be resulted from overtones of normal modes. Absorption bands between 3050 and 2850 cm^−1^ are due to carbonates in the mineral^23^. Water molecules are usually adsorbed by carbonate surfaces. Bands at 1623, 3403 and 3544 cm^−1^ are resulting from the bending and stretching modes of the adsorbed water^24^. In sum, Raman spectroscopy combined with FTIR analysis correlate well with our EDS/XRD analysis about the main presence of calcite mineral structure in the dust particles.

As our dust particles were found to contain a certain proportion of iron, we investigated if any magnetic property could be detected. The ultimate goal is to utilize these magnetic properties to design a mitigation process based on magnetic protection coating for the PV panels. Magnetic hysteresis loops contain a wealth of information and can be used to identify whether the magnetic mineral is soft or hard, and to identify the size of the magnetic particles^25^. A reference sample of a tape (that has no dust) of 80 mg was made to determine the susceptibility of the reference material and was found to be −5.4 × 10^−10^ EMU/Oe mg. A first rapid hysteresis loop with not many points was made to get an idea of the order of magnitude of the sample. The result showed that there was a very low coercivity and remanance combined with a high susceptibility on the test sample (Table 3), therefore many points were required between −2000 and 2000 Oe, as per the following:


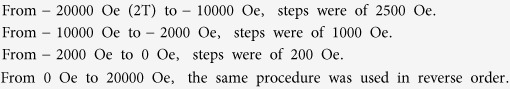


[Table t3]

**Figure 9:**
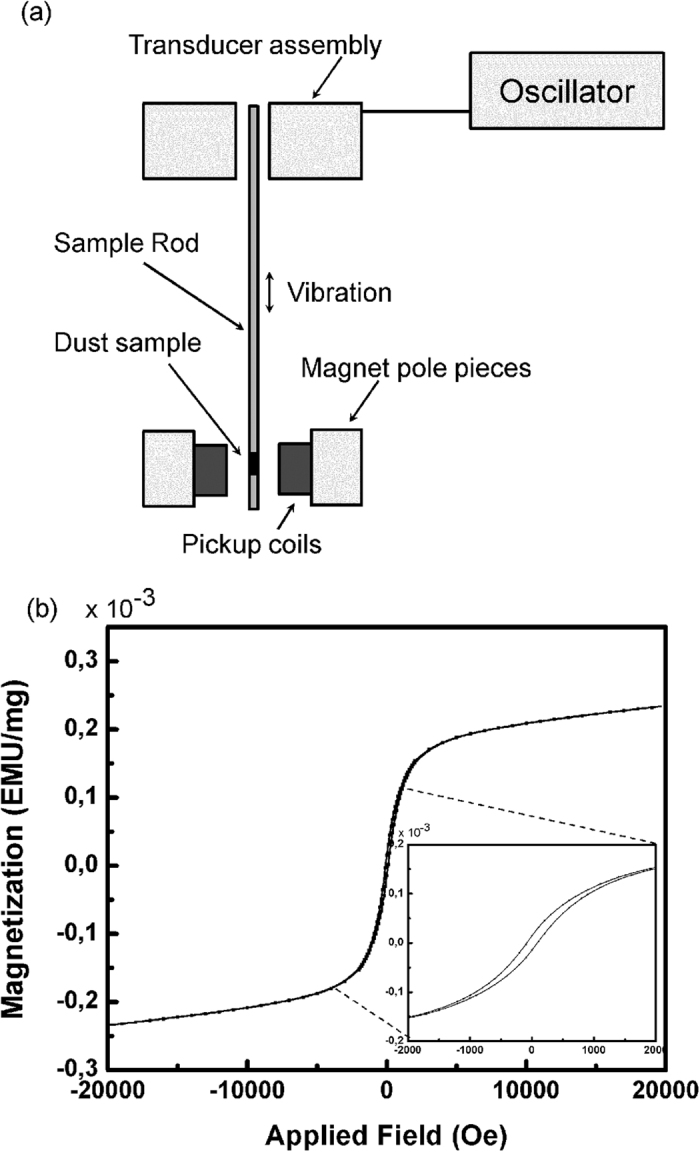
(**a**) Schematic of the VSM machine principle, (**b**) Magnetic hysteresis loop of dust particles.

Only magnetization measurements along the field axis were taken (Fig. 9a). From the hysteresis curve presented in Fig. 9b, several magnetic properties can be determined. The susceptibility was calculated from the slope of the linear region of the magnetization when the material was not saturated. This property gives the strength of the magnetic response of the material. The magnetic remanence is the magnetization of the material when there is no applied field. The stronger the remanence, the higher the field generated by the material on its own (without exterior field). The magnetic coercivity is the applied field necessary to cancel any remanence. A material with a high coercivity is considered “hard” (like permanent magnets), while a material with low coercivity is considered “soft” (like pure iron). Finally, the saturation field is the exterior field needed to saturate the material. This value is very approximate and corresponds to the beginning of saturation.

The shape of the hysteresis loop of dust particles in Qatar as shown in Fig. 9b closes under 700 mT (7000 Oe), indicating that the dominant magnetic minerals is soft (i.e. wuetite); however, magnetization still increases slightly with the applied field from 7000 to 10000 Oe, suggesting the possible presence of traces of hard magnetic minerals (i.e. hematite, goethite) which might not be present in significant amounts to be detected by the other mentioned characterization techniques.

The hysteresis loop shown in the inset of Fig. 9b shows narrow hysteresis loop which implies a small amount of dissipated energy. Narrow hysteresis loop has high permeability (slope of the magnetic momentum of magnetization to the applied magnetic field). These characteristics indicate that the source of magnetism in these particles is the presence of soft material. Another parameter to differentiate between soft and hard magnetic materials is the measurement of coercivity from the hysteresis loop. By analyzing Fig. 9b, it is estimated that coercivity = 92.38 Oe which is considered rather small, hence approving that the nature of wuestite is soft magnet. As a matter of fact, Ma *et al*.^25^ have compared the magnetic properties between a sample dust collected in Australia and other samples collected in China. They concluded that the magnetic minerals content of the Lanzhou dust-storm sample from China (LZD) is much higher than that of the Sydney sample from Australia (SDD).

**Figure 10:**
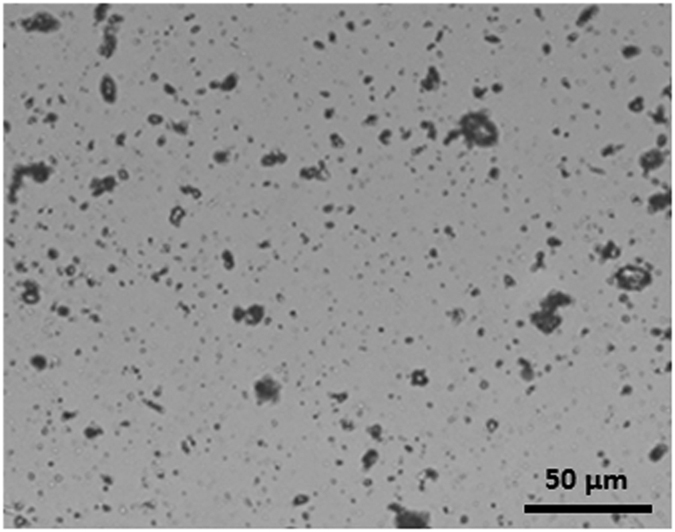
Optical microscope image of dust particles (40X) on a glass sample after one week exposure.

Finally, as all these characterizations aim at drawing a solid mitigation process based either on self-cleaning strategy or magneto-filtration, we present here the influence of the dust accumulation on the photovoltaic performance of the solar panels installed in the Solar Test facility in Doha (Qatar). Dust accumulation on the PV modules is resulting in a reduction of the power output by blocking the solar irradiance from reaching the PV cells. A typical example of an optical image of a dusty glass sample after one week exposure is shown in Fig. 10.

In this section, the dust effect on a multi-crystalline silicon PV module power output installed at the Solar Test Facility (Doha, Qatar) is reported.

**Figure 11:**
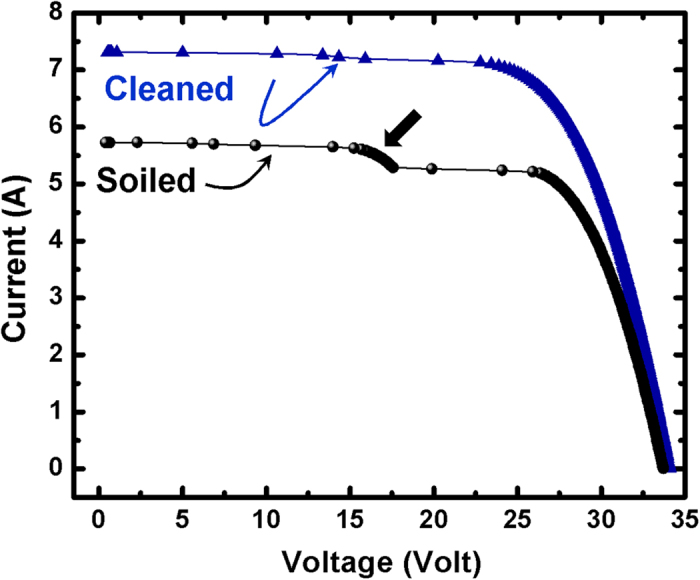
The IV curve measured at a given time for a single multi-crystalline silicon module before and after cleaning. An increase in the short current circuit *I*_*sc*_ was observed after module cleaning, while the short circuit voltage V_oc_ remains identical. A deviation of the IV curve on the soiled module is indicated by a black thick arrow.

Figure 11 shows the IV (current-voltage) curve measured for an individual solar module before and after cleaning. After module cleaning, the short current circuit *I*_*sc*_ increases, while the open circuit voltage *V*_*oc*_ remains quite similar. It is known that the photocurrent, and therefore the *I*_*sc*_, is mainly proportional to the solar irradiance. Therefore, dust accumulated on the solar module will prevent the solar irradiance from reaching the solar cells and causing light to reflect from the solar module. As a consequence, a drop in the *I*_*sc*_ was observed.

A deviation of the IV curve (indicated by black thick arrow in Fig. 11) was observed on the soiled module. This was also observed by different authors^26^,^27^,^28^ and could be explained by non-uniform shading on the front-glass surface of the module caused by soiling effect.

Table 4 summarizes the electrical parameters extracted from the IV curve. A clear increase in the short current circuit *I*_*sc*_ as high as 38% was measured after cleaning the PV module, which in turn results in an increase of 28% in the total power output. Since the module temperature measured remains within ±1 °C difference between the soiled and cleaned module, and the Global Plane of Array Irradiance (G-POA) increases by 7%, the increase in the *I*_*sc*_ could be explained only by removing the dust accumulated on the PV module where the effect of temperature could be excluded.

## Conclusion

In this paper, the structural and physical characteristics of Qatar dust particles collected from PV panels installed at the Solar Test Facility located at Doha city were investigated and their influences on the photovoltaic performance were evaluated. Various analytical tools including optical, scanning electron, X-ray diffraction, atomic force microscopy, Raman, FTIR, UV-Vis and VSM were used to characterize the dust particles. The main findings show that dust particles have mainly an average size of about 2 μm, in addition to the presence of larger non-uniform particles of few tens of micron size. EDS and X-ray diffraction analysis have shown that the particles are mostly composed of calcite mineral structure with about 58% atomic content as corroborated as well by Raman and FTIR spectra. To further investigate dust characteristics, the full hysteresis loop obtained by the vibrating sample magnetometer indicated the presence of magnetic properties in these dust particles typical to soft material. The effect of soiling on the optical transmittance properties of the glass substrate have shown a reduction of about 26% compared with the clean reference sample. Finally, the influence of dust particles accumulation on the solar panels was found to cause a clear drop in the PV power output. We believe that this original study could serve as a reference for the characteristics of the Qatar desert dust, and work is in progress to design a mitigation process based on these results.

## Additional Information

**How to cite this article**: Aïssa, B. *et al*. Structural and physical properties of the dust particles in Qatar and their influence on the PV panel performance. *Sci. Rep.* **6**, 31467; doi: 10.1038/srep31467 (2016).

## Figures and Tables

**Table 1:** Summary of the main chemical compounds of the dusty sample as identified by X-ray diffraction.

**Peak #**	**2-theta (**^**o**^)	**Phase chemical formula**
1	26.649	SiO_2_, Al_2_(SiO_4_)O, Mg_2_(SiO_4_)
2	29.3922	Ca(CO_3_)
3	30.93	Al_2_(SiO_4_)O, Ca_2_Mg(Si_2_O_7_)
4	35.9954	Ca(CO_3_), Ca_2_Mg(Si_2_O_7_)
5	37.3504	Al_2_(SiO_4_)O, Mg_2_(SiO_4_), Ca_2_Mg(Si_2_O_7_)
6	39.4116	Ca(CO_3_), SiO_2_, Al_2_(SiO_4_)O
7	41.0783	Al_2_(SiO_4_)O, FeO, Mg_2_(SiO_4_), Ca_2_Mg(Si_2_O_7_)
8	43.1698	Ca(CO_3_), Al_2_(SiO_4_)O
9	44.9443	Mg_2_(SiO_4_)
10	47.1611	Ca(CO_3_),
11	47.5344	Ca(CO_3_), Ca_2_Mg(Si_2_O_7_)
12	48.4681	Ca(CO_3_), Ca_2_Mg(Si_2_O_7_)
13	51.0323	SiO_2_, Al_2_(SiO_4_)O, Mg_2_(SiO_4_), Ca_2_Mg(Si_2_O_7_)
14	57.4174	Ca(CO_3_), Al_2_(SiO_4_)O, Mg_2_(SiO_4_)
15	64.658	Ca(CO_3_), Al_2_(SiO_4_)O, Ca_2_Mg(Si_2_O_7_)

**Table 2:** Most possible compositions of dust particles as suggested by XRD analysis interface software.

**Phase name**	**Chemical formula**	**Atomic content (%)**
Calcite	CaCO_3_	58
Quartz	SiO_2_	7
Sillimanite	Al_2_(SiO_4_)O	17
Wuestite	FeO	0.6
Olivine	Mg_2_(SiO_4_)	9
Akermanite	Ca_2_Mg(SiO_7_)	8

**Table 3 t3:** Summary of magnetic properties of Qatar’ dust particles.

**Magnetic property**	**Value**
Susceptibility (EMU/Oemg) × 10^−7^	9.5
Remanence (EMU/mg) × 10^−4^	4.3
Coercivity (Oe)	92.38
Approximate Saturation (Oe)	3000

**Table 4:** A comparison of the performance of a multi-crystalline silicon PV module before and after cleaning.

**Values**	***G-POA*****[W/m**^**2**^]	***T***_***mod***_**[°C]**	***I***_***mpp***_ **[A]**	***I***_***sc***_ **[A]**	***V***_***mpp***_ **[V]**	***V***_***oc***_ **[V]**	***P*****[W]**
Before cleaning (soiled)	884	41.3	5.14	5.726	26.76	33.72	137
After cleaning	944	40.7	6.73	7.312	26.19	34.08	176

*G-POA*: Global Plane of Array Irradiance, *T*_*mod*_: module temperature, *I*_*mpp*_: current at maximum power point, *I*_*sc*_: short circuit current, *V*_*mpp*_: voltage at maximum power, *V*_*oc*_: open circuit voltage and *P*: power output.
